# Special Issue: Genetic Basis of Autism Spectrum Disorder

**DOI:** 10.3390/ijms27104569

**Published:** 2026-05-19

**Authors:** Elisabetta Bolognesi, Franca Rosa Guerini

**Affiliations:** IRCCS Fondazione Don Carlo Gnocchi, 20148 Milan, Italy

Autism spectrum disorder (ASD) encompasses a wide and diverse range of neurodevelopmental conditions, characterized by difficulties with social communication along with restricted and repetitive patterns of behavior. Currently, ASD cannot be explained by a single framework based on a simple cause-and-effect relationship. However, advances in molecular genetics, high-throughput sequencing, and systems biology since the early 2010s have profoundly reshaped our understanding of the etiopathogenesis of ASD.

This Special Issue highlights emerging evidence of the multilayered biological architecture of ASD, emphasizing the role of rare and common genetic variants, sex-specific differences in immune-related factors, redox imbalance, and microbiota–brain interactions. Collectively, these findings suggest that ASD is not a uniform disorder but a heterogeneous condition arising from the convergence of multiple biological pathways affecting neurodevelopment.

In their comprehensive review, Nobrega et al. outline the progress in elucidating the genetic architecture of ASD through large-scale genomic studies and the establishment of extensive patient cohorts. Hundreds of genomic loci and over 100 high-confidence risk genes have been identified, underscoring the substantial contribution of genetic factors to the etiology of ASD. Rare de novo variants with high penetrance have been particularly informative in uncovering key biological pathways in ASD; however, these variants explain only a minority of cases. Conversely, common variants exert small individual effects, and the combined impact of common variants with environmental influences reflects a highly complex and still incompletely understood genetic landscape. Despite these advances, several major challenges remain in describing the genetic architecture of ASD. The interplay between rare and common variants has yet to be fully clarified, and polygenic risk scores currently lack sufficient predictive accuracy for routine clinical implementation. Similarly, copy number variants (CNVs), although among the most robust genetic findings in ASD, often span multiple genes, complicating the identification of gene-specific pathogenic mechanisms. The functional interpretation of noncoding variants also continues to pose substantial difficulties. Looking forward, further increases in cohort size, the continued refinement of genomic technologies, and the integration of multiomics datasets, including genomics, epigenomics, transcriptomics, proteomics, and metabolomics, are expected to accelerate advances in this field. Functional genomic approaches, such as CRISPR-based gene editing, stem-cell-derived neuronal systems, and brain organoids, together with advanced computational and machine learning strategies, will be instrumental in translating genetic findings into mechanistic insights. Ultimately, bridging gene discovery with neurobiological understanding will be essential for the development of precision medicine approaches tailored to biologically defined ASD subgroups.

Chromosomal abnormalities also illustrate the complexity of ASD. Gigonzac et al. report three patient cases involving chromosome 15q, a recurrent genomic region implicated in syndromic ASD [1]. Variants affecting 15q11.2–q13.1, 15q13.3, and 15q21.3–q26.2, including duplications, triplications, deletions, and uniparental disomy, highlight diverse pathogenic mechanisms from altered gene dosage to epigenetic dysregulation. Genes in this region, such as UBE3A, SNRPN, GABRB3, CHRNA7, and OTUD7A, are associated with epilepsy, intellectual disability, language impairment, and dysmorphic features, reflecting their pleiotropic effects. Both case studies [2] and population CNV analyses [3] reinforce the clinical heterogeneity linked to these variants and highlight the importance of comprehensive genomic profiling, chromosomal microarrays, methylation assays, and targeted validation for precision medicine and tailored therapeutic strategies.

Beyond classical neurodevelopmental genes, immune-related pathways appear to contribute to the susceptibility to ASD [4,5]. ASD is more frequently diagnosed in men, with a traditional male-to-female ratio of approximately 4:1 [6,7]. Recent large-scale cohort data from Sweden indicate that this ratio decreases over time and with increasing age at diagnosis, suggesting under or delayed diagnosis in women [8]. The presentations of women often diverge from the stereotypical male-based phenotype, and the ability of women to camouflage autistic traits may further obscure the recognition of ASD [9,10]. The concept of a female protective effect suggests that a larger accumulation of genetic and environmental risk factors may be required for ASD to manifest [11], potentially influenced by genetic and hormonal factors, including X-chromosome dosage.

In a cohort of Italian children with ASD, Guerini et al. analyzed HLA class I (HLA-A, -B, -Cw) and class II (HLA-DR) alleles, incorporating sex-stratified analyses. Specific alleles were found to act as sex-dependent risk or protective factors: HLA-A*02, B*38, and Cw*12 were more frequent in women with ASD, whereas HLA-B*44 was more prevalent in men with ASD. Conversely, HLA-A*03 was more commonly transmitted to healthy female siblings, suggesting a protective effect. These findings illustrate how immune-related genetic variation may intersect with neurodevelopment and contribute to sex differences in ASD vulnerability and resilience.

Environmental modifiers add further complexity to ASD. The gut–brain axis and the intestinal microbiota are key influencers of neurodevelopment [12]. Borbélyová et al. demonstrate that microbiota alterations can modulate behavior, synaptic function, intestinal permeability, and neuroendocrine signaling, particularly in genetically susceptible backgrounds such as Shank3 mouse models. SHANK genes encode postsynaptic scaffolding proteins critical for synaptic organization; SHANK3, in particular, supports cytoskeletal stability, neuritogenesis, and synaptogenesis, processes closely linked to ASD. Loss-of-function alterations in Shank3 result in mouse phenotypes that recapitulate key ASD-related traits, indicating that these models are particularly informative for mechanistic investigations. In their study, the fecal microbiota from children with ASD or normotypical controls was transplanted into SHANK3b+/− female mice. Wild-type male offspring whose parents received microbiota from ASD donors displayed delayed neurodevelopment. When combined with the Shank3b−/− mutation, fecal microbiota transplantation from ASD donors induced ASD-like behaviors in adult offspring, accompanied by increased gut permeability. Prenatal exposure to microbiota from children with ASD also impaired the postnatal regulation of food intake in Shank3b−/− male mice. These results highlight the influences of the interplay between genetic susceptibility and microbiota-driven environmental on neurodevelopment.

Oxidative stress pathways further contribute to ASD pathophysiology [13]. In this context, Spoto et al. investigated polymorphisms in genes encoding the phase I and phase II detoxification enzymes responsible for the metabolism of drugs and xenobiotics, as well as antioxidant enzymes in individuals with ASD compared with those of healthy controls. Spoto et al. also assessed stress biomarkers, including derivatives of reactive oxygen metabolites (dROMs), the biological antioxidant potential (BAP), and advanced oxidation protein products (AOPPs), and evaluated oxidative DNA damage. Mild oxidative stress was observed in patients with ASD relative to controls, with significantly higher AOPP levels and higher DNA damage in lymphocyte nuclei in those with ASD. When comparing patients ASD with their siblings, the difference diminished, suggesting a shared familial predisposition to oxidative stress that acts as a biological substrate upon which environmental triggers further operate. Moreover, associations were found between ASD and polymorphisms in genes involved in detoxification and oxidative stress responses. Overall, these findings support the hypothesis of a complex interplay between genetic susceptibility and environmental exposures in the etiology of ASD, with oxidative stress representing a potential mechanistic link.

Together, the studies collected in this Special Issue support a multifactorial view of ASD arising from intersecting genetic, epigenetic, immunological, metabolic, and environmental influences. This multidimensional perspective reflects the growing consensus that no single mechanism accounts for the heterogeneity of ASD and that approaches that combine multilayered omics data with detailed phenotypic characterization are needed to advance the field.

Future directions in ASD research should include expanding genomic research to under-represented populations, integrating multiomics data with functional validation, standardizing sex-specific analyses, and translating molecular discoveries into clinical tools, such as biomarkers, risk stratification methods, and targeted therapies. In this context, machine learning and artificial intelligence approaches are expected to play a key role in integrating and interpreting complex, high-dimensional datasets, thereby supporting the identification of biologically and clinically meaningful patterns. By embracing the complexity of ASD, the field can move toward precision medicine strategies that ultimately enhance diagnosis, counseling, and personalized interventions for affected individuals.
